# Abstract Representations of Sensorimotor Transformations in Human Premotor Cortex

**DOI:** 10.64898/2026.08.12.744241

**Published:** 2026-10-16

**Authors:** Sanghoon Kang, Juliana E. Trach, Samuel D. McDougle

## Abstract

Flexible motor control requires representations of context-specific mappings that connect goals to actions. If and how sensorimotor cortical areas encode these mappings is poorly understood. Here, we asked if the human sensorimotor cortex, in addition to encoding movements, also encodes latent mappings between visual goals and movements. Participants learned to adapt wrist movements under two distinct visuomotor transformations, controlling a visual cursor that was either rotated or reflected away from their hand movement. Representational similarity analyses on fMRI data dissociated neural activity patterns related to transformation context, movement direction, and target location. We observed distinct representational profiles within sensorimotor cortex: Premotor cortex activity patterns encoded both movement direction and transformation context, whereas primary motor cortex activity patterns encoded movement direction but not transformation context. In dorsal premotor cortex, stronger transformation context encoding was associated with better task performance. Moreover, in cases where the two transformations converged on the same movement solution, which permitted retrieval of a cached target-response association, participants with stronger behavioral signatures of caching showed greater overlap between transformation and movement representations in dorsal premotor cortex. These findings suggest that premotor cortex flexibly represents abstract sensorimotor transformations linking goals to actions, providing a neural interface between action selection strategies and motor commands.

## Full Text Availability

The license terms selected by the author(s) for this preprint version do not permit archiving in PMC. The full text is available from the preprint server.

